# Altered DNA Methyltransferase Expression in Pulmonary Large‐Cell Neuroendocrine Carcinoma: Pilot Experimental Data Targeted DNMT1, DNMT3A, and DNMT3B

**DOI:** 10.1002/cnr2.70513

**Published:** 2026-03-19

**Authors:** Adam Put, Katerina Smesny Trtkova, Marcel Mittak, Petra Herentinova, Jana Vaculova, Radoslava Cernekova, Jozef Skarda

**Affiliations:** ^1^ Department of Clinical and Molecular Pathology and Medical Genetics, Faculty of Medicine University of Ostrava Ostrava Czechia; ^2^ Department of Clinical and Molecular Pathology and Medical Genetics University Hospital Ostrava Ostrava Czechia; ^3^ Department of Clinical and Molecular Pathology, Faculty of Medicine Palacký University Olomouc Olomouc Czechia

**Keywords:** DNA methyltransferases, DNMT3B isoforms, large cell neuroendocrine carcinomas

## Abstract

**Introduction:**

Large cell neuroendocrine carcinoma (LCNEC) is a subtype of non‐small‐cell lung carcinoma with poorly understood methylation characteristics, including the activity of DNA methyltransferases. Despite the pivotal role of DNA methyltransferases in epigenetic regulation, their expression profile in LCNEC remains unexplored. This study represents the first effort to evaluate the expression of *DNMT1*, *DNMT3A*, and *DNMT3B* genes in patients with LCNEC.

**Materials and Methods:**

We performed quantitative expression analyses of DNMT1, DNMT3A, and DNMT3B on formalin‐fixed, paraffin‐embedded tissue samples obtained from LCNEC patients. Normal lung tissues (*n* = 4) from healthy patients served as controls to determine differential expression levels.

**Results:**

The analysis revealed upregulation of both DNMT1 and DNMT3A compared to control normal lung tissue. In addition, normalized expression values of DNMT3B were reduced compared to the analyzed DNMT1 and DNMT3A.

**Conclusion:**

In this exploratory pilot of 18 LCNEC cases, DNMT1 and DNMT3A transcripts were frequently higher relative to a normal‐lung calibrator, while DNMT3B results were inconclusive due to technical limitations and small sample size. These descriptive findings warrant validation in larger, isoform‐resolved cohorts before clinical inference. Future research should prioritize investigating these DNA methyltransferases to explore their therapeutic implications. Moreover, future research should also aim to detect and characterize individual DNMT3B isoforms in LCNEC patients to further elucidate their specific contributions to the pathology of the disease.

## 1 Introduction

Lung carcinoma is a heterogeneous disease, representing the leading cause of cancer‐related deaths globally. In both sexes combined, lung cancer is the most commonly diagnosed cancer (11.6% of total cases) and the leading cause of cancer death (18.4% of total cancer deaths) [1]. Lung cancers are divided into non‐small cell carcinoma (NSCLC), comprising 80%–85% cases, and small cell carcinoma (small cell lung carcinoma, SCLC), which accounts for 15%–20% cases. Major types of NSCLC include adenocarcinoma, squamous cell carcinoma (SSC), and large cell carcinoma (LCC) [2]. LCCs represent a minority of NSCLCs (less than 3% of the lung cancers) and lack morphologic and immunohistochemical evidence of adenocarcinoma, SCC, or neuroendocrine carcinoma. The large cell neuroendocrine carcinoma (LCNEC) is classified in the lung neuroendocrine tumors that are relatively infrequent, accounting for about 3% of lung cancers [3, 4].

Gene expression in lung cancer can be disrupted not only by mutations in tumor suppressor genes (TSGs) and oncogenes but also by epigenetic modifications during the early stages of cancer progression [5]. These epigenetic alterations, including DNA methylation, non‐coding RNA expression, and histone modification, play a role in modulating gene transcription by altering the accessibility of DNA to transcription factors. DNA methylation is among the most extensively studied epigenetic modifications and is implicated in various processes such as genomic imprinting, mammalian development, and cancer progression [5, 6]. In lung tumorigenesis, DNMT upregulation disrupts the delicate balance between cell proliferation and differentiation, driving uncontrolled growth and tumor formation [7]. Specific protein interactions stabilize DNMT during tumorigenesis, further inhibiting cell differentiation and enabling tumor cells to grow independently of external stimuli [8]. Although surgery is the primary treatment for early‐stage NSCLC, the majority of patients are diagnosed at an advanced stage, where curative resection is not an option. DNA methylation markers, such as those found in genes like *APC*, *HOXA9*, *RARβ2*, and *RASSF1A*, have been proposed as potential tools to differentiate between lung cancer subtypes, particularly SCLC and NSCLC [9].

Future therapeutic strategies may leverage epigenetic approaches, such as DNA methyltransferase inhibitors (DNMTis), in treating lung cancer. DNMTis like 5‐Azacytidine (5‐AZA) and its derivative 5‐Aza‐2′‐deoxycytidine (DAC, decitabine) work by forming covalent bonds with DNA methyltransferases (DNMTs), leading to enzyme inactivation. These drugs, however, face limitations such as chemical instability, low bioavailability [10, 11], and off‐target effects stemming from non‐selective inhibition, which constrain their application in solid tumors [12]. To surpass these challenges, more stable nucleoside analogs, such as SGI‐110 (Guadecitabine), and non‐nucleoside compounds are being explored [13, 14]. Furthermore, a highly potent and selective small‐molecule DNMT1 inhibitor, GSK3484862, has been developed [15]. Combination therapy, where cancer cells are first primed with an epigenetic drug before exposure to chemotherapy, is also a promising avenue. A phase I clinical trial investigating the combination of decitabine with the histone deacetylase inhibitor (HDACi) valproic acid demonstrated promising outcomes in patients with advanced NSCLC (p5) [16]. Additionally, the novel epigenetic agent RRx‐001, which targets HDACs, DNMT1, and DNMT3A, is currently undergoing evaluation in combination with platinum‐based chemotherapy [17].

DNA methyltransferases mediate the transfer of methyl groups to cytosines in CpG dinucleotides, primarily within CpG islands located in over half of human gene promoters. Cancer cells often exhibit two types of DNA methylation abnormalities: hypomethylation and hypermethylation. Promoter hypermethylation can result in the silencing of tumor suppressor genes and other genes essential for cancer progression, including those involved in metastasis, invasion, and immune responses, such as mismatch repair genes (*MLH1* and *MSH2*) [18], homologous recombination repair genes (*BRCA1* and *RAD51*), the TERT oncogene, and genes within the *CDKN2B*/*CDKN2A*/*RB1*/*E2F* pathway. Furthermore, global DNA hypomethylation, which occurs during aging or cellular senescence, is a hallmark of aggressive cancer and is associated with genomic instability [19].

DNA methyltransferases (DNMTs) consist of a C‐terminal catalytic domain and an N‐terminal regulatory domain. In humans, five DNMTs are known, each exhibiting varying specificities for methylated and unmethylated DNA: DNMT1, DNMT2, DNMT3A, DNMT3B, and DNMT3L. DNMT1, also known as the maintenance methyltransferase, preserves methylation patterns during DNA replication. DNMT3A and DNMT3B, referred to as de novo methyltransferases, are primarily expressed in undifferentiated cells and are essential for establishing and maintaining new DNA methylation patterns. Although DNMT3L lacks a catalytic domain, it interacts with DNMT3A and DNMT3B (specifically DNMT3B1 and DNMT3B2) to enhance their catalytic activity [20]. Deletions in the C‐terminal region of DNMT3B may disrupt its interaction with DNMT3L, as evidence indicates that the C‐terminal catalytic domain of DNMT3B1 is critical for its association with DNMT3L.

Aberrant DNA methylation is associated with many malignancies, including lung cancer. Lung cancer is one of the most common malignant tumors in which the morbidity and mortality rates are nearly the same. Therefore, how to detect early lung cancer effectively and accurately has become a difficult problem for physicians. The LCNEC is a highly aggressive and rare subtype of lung cancer for which standardized treatment protocols are currently lacking. Differences in methylation patterns have been observed across lung cancer subtypes, and multiple epigenetic factors—in particular, DNA methylation—have been associated with the development of various types of lung cancers [12]. In the age of molecular diagnosis, DNA methylation is very essential to solving these problems.

According to epidemiological statistics, LCNEC is closely related to smoking, second‐hand smoke exposure, and most patients with SCLC have a history of smoking [21]. The markers of methylation caused by smoking persist years after quitting smoking, and several scientists have found that DNA methylation is a “bridge” between smoking and lung cancer [8]. The aim of our pilot study was to determine the expression profile of three DNMTs, as their enzymatic DNA‐methyltransferase activity is essential for influencing other molecules of signaling pathways, such as PI3K/AKT/mTOR, leading to or supporting tumor cell proliferation.

Furthermore, the DNMTs expression could emerge as a target marker in lung cancer, supporting the potential use of DNMTis as a treatment strategy. To date, DNMTs expression has not been systematically evaluated in LCNEC, and despite many reports about the involvement of DNMTs in human cancers, including.

## 2 Materials and Methods

### 2.1 Materials

Set of 18 patient paraffin‐embedded tissue samples (FFPET) samples with large‐cell neuroendocrine carcinoma, and separate FFPET sample of normal lung tissue were provided from archival sources of Department of Clinical and Molecular Pathology and Medical Genetics of University Hospital Ostrava. The age range of the patients at the time of diagnosis was 48–77 years (median 66 years). Patients included in this study had a confirmed histopathological diagnosis of LCNEC based on the World Health Organization (WHO) classification, supported by immunohistochemical markers such as chromogranin A, synaptophysin, and CD56. Clinicopathological data are provided in Supporting Information (Table S1) sufficient FFPET was available for molecular or histological analysis. Only adult patients aged 18 years or older were included, regardless of disease stage, and both treatment‐naïve patients and those who had received standard therapies were eligible. Patients were excluded if they had insufficient tumor tissue for analysis, non‐LCNEC histological subtypes, or active malignancies other than LCNEC. Informed consent was obtained from all patients prior to sample collection, in accordance with the principles outlined in the Declaration of Helsinki and approved by the appropriate Institutional Ethics Committee (IEC) University Hospital Ostrava.

### 2.2 Reverse Transcription‐Quantitative PCR (RT‐qPCR)

Total RNA was extracted from a single FFPET section with a maximum thickness of 10 μm, using the High Pure FFPET RNA Isolation Kit (Roche, Basel, Switzerland). Reverse transcription of 300 ng of total RNA was performed with the One Script Plus cDNA Synthesis Kit (Applied Biological Materials Inc., Richmond, BC, Canada). RNA purity (260/280 and 260/230 ratios) was measured by NanoDrop One Microvolume UV–Vis Spectrophotometer (Thermo Scientific). We then performed agarose gel electrophoresis to assess RNA integrity and confirm that the samples were suitable for downstream qRT‐PCR analysis. Quantitative real‐time PCR was conducted with TaqMan probes using the Xceed qPCR Probe Mix (Institute of Applied Biotechnologies, Cincinnati, Ohio, USA) on the LightCycler 480 System (Roche). Expression levels of DNMT1, DNMT3A, and DNMT3B were normalized to the endogenous reference gene beta‐2 microglobulin (B2M). TaqMan probes for all genes were obtained from Thermo Fisher Scientific (Waltham, Massachusetts, USA) with following assay ID: B2M (Hs00187842_m1), DNMT1 (Hs00945875_m1), DNMT3A (Hs01027162_m1), and DNMT3B (Hs00171876_m1) (Table S2). A control FFPET sample of normal lung tissues (*n* = 4) served as the calibrator for the comparative Ct method (2‐∆∆Cq) to quantify the relative expression of the target genes. Mean ΔCq values with 95% confidence intervals (CI) for DNMT1, DNMT3A, and DNMT3B are shown in (Table S3). All experiments were performed in triplicate, with three independent replicates.

### 2.3 Statistical Analysis

Descriptive statistics were applied to summarize gene expression and clinicopathological data. Expression values were analyzed both categorically (upregulated expression > 0, downregulated expression < 0) and as continuous variables. Group distributions were reported as absolute numbers and percentages. Associations between categorical clinicopathological features (sex, smoking status, recurrence) and expression status were explored using contingency tables. Continuous variables (age at diagnosis, tumor diameter) were analyzed descriptively (mean, range).

To assess potential associations between continuous expression values (DNMT1, DNMT3A, DNMT3B) and clinicopathological data (age, tumor size, peritumoral inflammation), correlation analyses were performed using Pearson's correlation coefficient and Spearman's rank correlation coefficient. Scatterplots with regression lines were generated for visualization. Given the limited sample sizes and especially the strong imbalance in expression categories for DNMT1 and DNMT3A, as well as the very small sample for DNMT3B, all analyses were interpreted in an exploratory manner. All statistical analyses and data visualizations were performed in Python (pandas, scipy, seaborn, matplotlib).

Publicly available RNA‐seq datasets were used for in silico validation. Normal lung transcriptomes were obtained from GTEx v10 (“Gene TPMs by tissue – Lung;” *n* = 604). LCNEC RNA‐seq data were extracted from the cohort published by George et al. (2018; Supporting Information 11; *n* = 66). Expression values (TPM for GTEx and RSEM expected counts for LCNEC) were harmonized by log2(x + 1) transformation and analyzed in R/Python. Distributions were compared descriptively using Cohen's d effect sizes and Mann–Whitney U tests, acknowledging platform and pipeline differences. Boxplots were generated in Matplotlib with median, interquartile range, and 1.5 × IQR whiskers.

## 3 Results and Discussion

**FIGURE 1:**
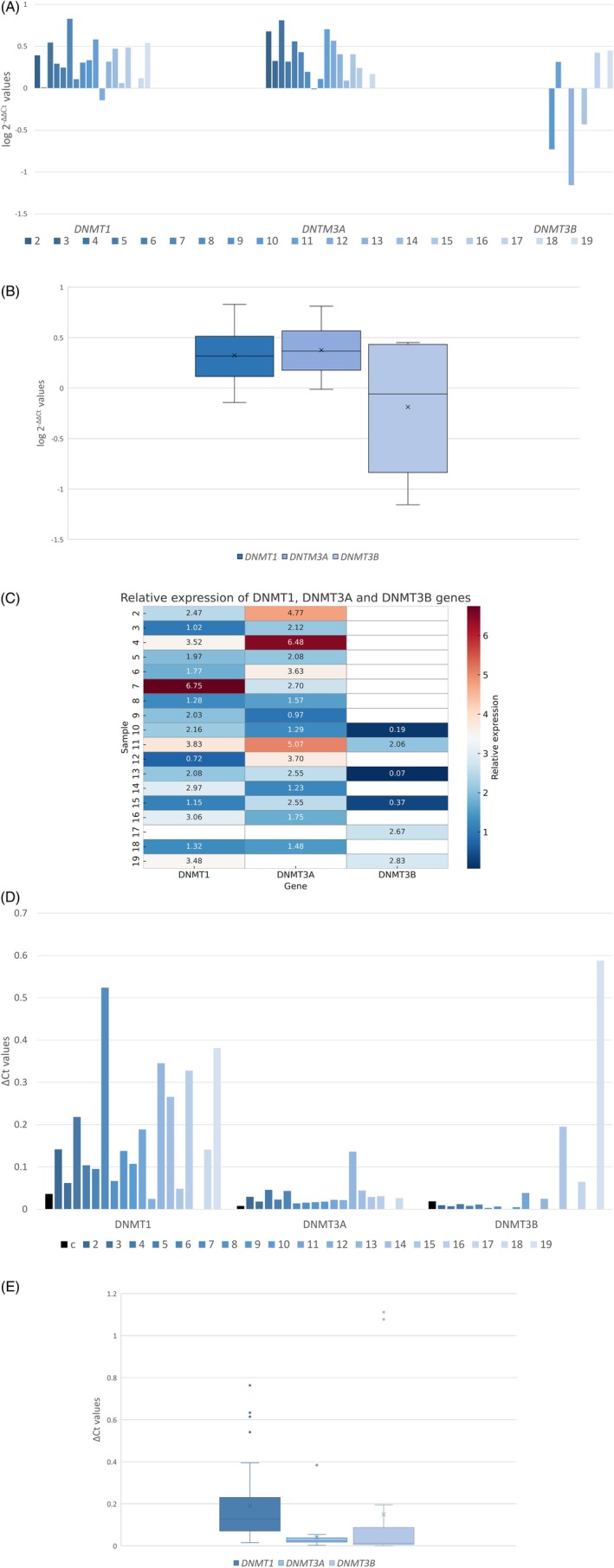
Comparison of log 2^‐∆∆Cq^ values (A, B) and ∆Cq values (D, E) of *DNMT1*, *DNMT3A* and *DNMT3B* genes was performed in LCNEC patient samples. (A) Column graph illustrates the logarithmic mean relative expression for each patient sample while (B) presents a boxplot of all values for each DNA methyltransferase. Log 2^‐∆∆Cq^ values > 0 indicate increased expression, values < 0 indicate decreased expression, and values ~0 indicate unchanged expression. (C) Heatmap illustrates comparison between DNMT1, DNMT3A and DNMT3B within each patient sample (relative expression values > 1 indicate increased expression, values < 1 indicate decreased expression, and values ~1 indicate decreased expression). (D) Column graph shows the absolute expression values for each patient sample with control columns marked as black, and (E) presents a boxplot summarizing expression values for each DNA methyltransferase. DNMT1/3A/3B—DNA methyltransferase 1/3A/3B; LCNEC—large cell neuroendocrine carcinoma.

Upregulation of DNA methyltransferases (DNMTs), particularly DNMT1, was frequently observed in NSCLC and was consistently associated with poor prognosis [22]. In our cohort of 18 patients diagnosed with LCNEC, relative quantification revealed overexpression of DNMT1 and DNMT3A, as well as heterogeneous expression of DNMT3B (Figure 1A,B). The levels of DNMT1 and DNMT3A expression were positively correlated in most patient samples (Figure 1C). An increased ∆Cq value was observed for the DNMT1 gene, whereas the ∆Cq values of DNMT3A and DNMT3B were comparatively lower (Figure 1D,E).

In line with these findings, our statistical analysis confirmed that DNMT1 and DNMT3A were predominantly overexpressed (16/17 and 15/16 patients, respectively), while DNMT3B displayed a balanced distribution (3/6 upregulated expression vs. 3/6 downregulated expression). Continuous expression values correlated with clinicopathological parameters showed no statistically significant associations (Table S4). For DNMT1, a weak positive correlation with age was noted (Pearson *r* = 0.39, *p* = 0.13) (Figure S5), whereas tumor size (*r* = −0.17, *p* = 0.53) (Figure S6) and peritumoral inflammation (*r* = 0.05, *p* = 0.86) showed no relationship. DNMT3A expression similarly lacked correlations with age (*r* = −0.09, *p* = 0.75) (Figure S7), tumor size (*r* = 0.07, *p* = 0.79) (Figure S8), and inflammation (*r* = −0.14, *p* = 0.62). For DNMT3B, no associations were detected with age (*r* = −0.17, *p* = 0.74), tumor size (*r* = 0.01, *p* = 0.98), or inflammation (*r* = 0.21, *p* = 0.69).

To contextualize our qPCR findings, we compared DNMT1/3A/3B/3 L expression in an independent LCNEC RNA‐seq cohort [23] (*n* = 66) with normal lung from GTEx (v10; *n* = 604). Both datasets were transformed into log2(x + 1). LCNEC displayed markedly higher distributions of DNMT1, DNMT3A, and DNMT3B relative to normal lung (Cohen's d 13–18; Mann–Whitney *p* < 10^−9^), whereas DNMT3L was near‐undetectable in both cohorts (median = 0). These cross‐cohort results support elevated DNMT1/3A/3B expression in LCNEC (Figure S9).

Given the frequently detected overexpression of DNMT1 and DNMT3A in our LCNEC cohort, these patients may represent suitable candidates for therapies targeting DNA methyltransferases. Hypomethylating agents, such as 5‐AZA or decitabine, could potentially reverse aberrant methylation patterns and sensitize tumors to standard chemotherapy or radiotherapy [24, 25]. Our findings therefore provide additional rationale for exploring DNMTis as a therapeutic option in LCNEC, a disease entity currently lacking standardized treatment approaches.

Importantly, DNMT1 overexpression has been linked to poor prognosis, and its prognostic significance was particularly evident in patients with squamous carcinoma. Kim et al. detected elevated expression of DNMT1 in NSCLC patients (22/34 adenocarcinoma, 25/53 squamous cell carcinoma, 7/15 others—not specified), by comparison to matched normal lung tissue from each patient [22]. Moreover, Kwon et al. found higher expression of DNMT1 in NSCLC patients (14/73 squamous cell carcinomas, 25/59 adenocarcinomas and 8/21 others—large‐cell carcinomas and typical carcinoids) compared to matched normal tissue, that was associated with poor differentiation exposure to tobacco smoke in adenocarcinoma [26]. The overexpression and strong binding of various DNMTs can lead to the promoter hypermethylation of key tumor suppressor genes coding FHIT, p16(INK4a), and RARβ proteins, contributing to lung tumorigenesis and poor prognosis [27].

Furthermore, Liu et al. (2016) found inhibition of DNMT1 expression in human lung adenocarcinoma cell lines. Reduced DNMT1 levels, both at the transcript and protein levels, in response to epigenetic therapies were associated with treatment response in mice. These findings support the potential for developing DNMT1‐targeted therapeutic strategies [28]. Treatment of 5‐AZA may have the potential to reverse the metastasis‐prone signature, as was shown in aggressive phenotype NSCLC cells [29]. DNMTis also induce a BRCAness phenotype in NSCLC (NSCLC with BRCA mutations) by downregulating key DNA repair genes, enhancing the sensitivity of NSCLC cells to PARP inhibitors and ionizing radiation both in vitro and in vivo [30].

Specifically, DNMT3B has nearly 40 alternative spliced variants of DNMT3B [31]. Members of the DNMT3B subfamily exhibit tissue‐specific expression, with the DNMT3B gene encoding multiple isoforms that fall into three categories mainly by which part of DNMT3B is lacking: (1) DNMT3B3–7 lacked the C‐terminal domain of DNMT3B; (2) DNMTΔ3B family lacked the N‐terminal domain of DNMT3B [32, 33]; and (3) DNMT3B∆5 and DNMT3B∆(4 + 5) lacked some amino acid residues close to PWWP domain of DNMT3B [34]. However, in our study, both ∆Cq and 2‐∆∆Cq values of DNMT3B were difficult to established due to the inability to determine *DNMT3B* gene expression in non‐cancerous lung tissue. Furthermore, the available detection DNMT3B probe (Hs00171876_m1) we used, did not consider the specificity of the isoforms described above [33, 35, 36]. The Hs00171876_m1 probe interrogates sequences of transcripts located in the boundaries of exons 5–6 and 7–8 and determines multiple types of isoforms: DNMT3B1 (NP_008823.1), DNMT3B2 (NP.787044.1), DNMT3B7 (NP_001193984.1), DNMT3B8 (NP_001193985.1), DNMT3B18 (NP_001411289.1). We were unable to detect for example isoforms DNMT3B∆3, DNMT3B∆5, and DNMT3B∆(4 + 5) which lacked some amino acid residues close to PWWP domain. Therefore, the complexity of isoforms, especially of DNMT3B, may not have been fully captured by our qPCR assay and should be acknowledged as a limitation. It is obvious that members of the DNMTΔ3B subfamily could play a significant role in the development of aberrant promoter methylation during lung tumorigenesis.

The DNMTΔ3B subfamily represents a predominant transcript of DNMT3B in non‐small cell lung carcinoma, with frequent expression observed in primary NSCLC tumors but minimal or undetectable expression in corresponding normal lung tissue [33]. This finding may be a reason for our difficult DNMT3B detection in control normal lung tissue sample, and the consequent impossibility of determining the normalized/relative *DNMT3B* gene expression in almost all analyzed samples (Figure 1A–C). The DNMTΔ3B subfamily contains 7 isoforms (Figure S10A,B): DNMTΔ3B1—DNMTΔB4 are catalytically active DNMTs while DNMTΔ3B5—DNMTΔ3B7 lack the catalytic domain [28, 29]. Wang et al. evaluated the expression of ΔDNMT3B variants in NSCLC patients compared to corresponding normal lung tissue. Most observed overexpression was in variants ΔDNMT3B1 and ΔDNMT3B2 in NSCLC (36/45 adenocarcinomas and 29/37 squamous cell carcinomas). While the overexpression of ΔDNMT3B4 (25 adenocarcinomas and 25 squamous cell carcinomas) strongly correlates with *RASSF1A* promoter gene methylation, the overexpression of ΔDNMT3B5/6/7 (9/13/5 adenocarcinomas and 10/16/11 squamous cell carcinomas) correlates with poor clinical outcomes [33].

DNMT3L is essential for the establishment of DNA methylation patterns in germ cells. While the human DNMT3L gene lacks intrinsic enzymatic activity, it encodes a 387‐amino‐acid protein characterized by a cysteine‐rich region with a unique zinc finger domain. This protein is expressed exclusively in embryonic stem cells and germ cells [20, 36, 37]. DNMT3L is distributed throughout the nucleus and cytoplasm in embryonic stem (ES) cells deficient in both DNMT3A and DNMT3B. However, its localization is restored upon ectopic expression of DNMT3A2, though not DNMT3A or DNMT3B. Additionally, specific CpG sites were found to be demethylated in ES cells when either DNMT3A or DNMT3L was depleted, but not when DNMT3B was depleted [38]. Although in our study, we did not quantify DNMT3L expression, it is likely that a potential cooperation between DNMT3A2 isoform and members of the DNMT3B (DNMT3B1 or DNMT3B2) or DNMTΔ3B subfamilies could lead to increased relative DNMT3A expression as a result of increased methylation activity of this gene.

The present study has several limitations that should be acknowledged. First, the low number of tumor tissue samples analyzed due to the rarity of LCNEC, which poses significant challenges in obtaining sufficient sample sizes for robust statistical analyses. Second, the absence of comprehensive clinicopathological data for several patients further limits the ability to correlate DNMT expression with clinical outcomes. These limitations constrain the generalizability of the findings and underscore the need for future research involving a larger cohort of LCNEC patients with complete clinicopathological data. And third, the probable reason for the difficult‐to‐detect expression of the *DNMT3B* gene in this type of lung tumors is probably isoform diversity and a possible mismatch between the commercial assays used vs. DNMT3B isoforms. Such studies would enable more definitive conclusions and facilitate the identification of reliable prognostic and therapeutic biomarkers for this aggressive lung cancer subtype.

The in silico validation integrates independent cohorts generated with distinct pipelines (LCNEC RSEM expected counts vs. GTEx TPM). Therefore, results are descriptive and may be influenced by batch effects. GTEx represents normal lung tissue from non‐cancer donors, not matched adjacent lung. Moreover, DNMT3B isoform heterogeneity is not resolved by this approach.

In summary, our study demonstrates frequent overexpression of DNMT1 and DNMT3A in patients with large‐cell neuroendocrine carcinoma, while DNMT3B expression was heterogeneous. Although no significant correlations between DNMT expression levels and clinicopathological variables were detected in this limited cohort, the consistent overexpression of DNMT1 and DNMT3A indicates their potential role in LCNEC pathogenesis. Given the lack of standardized treatment options for LCNEC and the evidence linking DNMT activity to tumor suppressor gene silencing, patients with DNMT1 and DNMT3A overexpression may benefit from therapies targeting DNA methyltransferases.

DNMTis directly impact DNA methylation at a global level, whereas other epigenetic therapies, such as HDACi and enhancer of zeste homologue inhibitors (EZH2i), can have indirect effects on DNA methylation or gene expression, and are thus often explored in combination with DNMTis [39]. As a monotherapy, 5‐AZA and decitabine are cytidine analogues that incorporate into DNA and inhibit DNMT activity, leading to degradation by the proteasome and consequently resulting in hypomethylation during cell replication. However, to date no clinical trials have been undertaken with the new selective non‐nucleoside compounds.

The use of epigenetic modulators in multidrug combination therapies has been shown to improve cancer treatments and potentially overcome drug resistance. Synergy between epigenetic drugs and immunotherapies could be a promising area for cancer treatment, as demonstrated by results from phase I and II trials, where the combination of nucleoside‐based DNMTis with other epigenetic drugs, immunotherapies, or chemotherapy resulted in potential benefit in NSCLC [39, 40]. Therefore, in a potential preclinical model, our findings could serve as rational targets for further investigation of the effect of DNMTis as monotherapy or strategies in combination with other epigenetic modulators in patients with LCNEC.

Future research should focus on larger patient cohorts with comprehensive clinicopathological data and should incorporate isoform‐specific analyses, particularly for DNMT3B, whose alternative transcripts may contribute to differential methylation activity and clinical outcomes. Such studies will be critical to validate DNMTs as biomarkers and to establish their utility as therapeutic targets in this rare and aggressive lung cancer subtype.

## Author Contributions

**Adam Put:** writing – review and editing (lead), writing – original draft (lead), visualization (equal), validation (equal), conceptualization (equal). **Katerina Smesny Trtkova:** writing – review and editing (lead), writing – original draft (lead), conceptualization (equal), visualization (equal), investigation (lead), formal analysis (equal), project administration (lead). **Marcel Mittak:** writing – review and editing (equal), resources (lead), conceptualization (equal). **Petra Heretinova:** writing – review and editing (equal), methodology (lead), formal analysis (equal). **Jana Vaculova:** writing – review and editing (equal), validation (equal), supervision (equal), investigation (equal). **Radoslava Cernekova:** writing – review and editing (equal), resources (equal), investigation (equal). **Jozef Skarda:** writing – review and editing (equal), validation (equal), supervision (lead), resources (equal), funding acquisition (lead).

## Funding

This article has been produced with the financial support of the European Union under the LERCO project number CZ.10.03.01/00/22_003/0000003 via the Operational Program Just Transition. This work was also supported by Palacký Univerzity Olomouc grant IGA LF 2024_010.

## Ethics Statement

This study was reviewed and approved by the University Hospital Ostrava Institutional Review Board (243/2025).

## Consent

Written informed consent was obtained from all the participants.

## Conflicts of Interest

The authors declare no conflicts of interest.

## Supporting information


**Figure S5.** Scatterplot of DNMT1 expression versus patient age. A non‐significant positive correlation was observed, suggesting a trend toward higher DNMT1 expression with increasing age.

**Figure S6.** Scatterplot of DNMT1 expression versus tumor diameter. No correlation was found between DNMT1 expression levels and tumor size.

**Figure S7.** Scatterplot of DNMT3A expression versus patient age. No correlation was observed between DNMT3A expression levels and patient age (Pearson r = −0.09, *p* = 0.75).

**Figure S8.** Scatterplot of DNMT3A expression versus tumor diameter. No correlation was observed between DNMT3A expression and tumor size (Pearson *r* = 0.07, *p* = 0.79).

**Figure S9.** Expression distributions of DNMT1, DNMT3A, DNMT3B, and DNMT3L in LCNEC (George et al. 2018; RNA‐seq, RSEM expected counts; *n* = 66) versus normal lung (GTEx v10; TPM; *n* = 604). Values were log2(x + 1) transformed; boxes show IQR with median; whiskers depict 1.5 × IQR, outliers hidden. Because of different pipelines (RSEM vs. TPM), this comparison is descriptive.

**Figure S10.** Schematic diagram of some members DNMT3B (3B1, 3B2, 3B3, 3B4, 3B5) and ΔDNMT3B (Δ3B1, Δ3B2, Δ3B3, Δ3B4, Δ3B5, Δ3B6, Δ3B7) subfamilies, and DNMT3L (A) and a comparison their transcripts (B). Figure adapted from Gujar et al. 2019, *Genes*, with modifications [29]. DNMT3B/3 L—DNA methyltransferase 3B/3 L.

**Table S1:** Clinicopathological features of the 18 cases of LCNEC.
**Table S2:** Table of primers used for quantitative Real‐time PCR.
**Table S3:** Mean ΔCq values with 95% confidence intervals (CI) for DNMT1, DNMT3A, and DNMT3B expression level relative to the calibrator sample. Values represent point estimates (mean differences) against the calibrator.
**Table S4:** Summary of DNMT1, DNMT3A, and DNMT3B expression and their associations with clinicopathological parameters in lung cancer patients. Patients were categorized as having upregulated expression (> 0) or downregulated expression (< 0) of each gene. Values are shown as counts, percentages, means, or correlation coefficients (Pearson's r, *p*‐values). Overexpression predominated for DNMT1 and DNMT3A, while DNMT3B displayed a balanced distribution. No statistically significant correlations between gene expression and clinicopathological parameters were observed, although DNMT1 expression showed a weak, non‐significant trend toward higher levels with increasing age.

## Data Availability

The data that support the findings of this study are available from the corresponding author upon reasonable request.
