# Supplementary figures and images for: Altered DNA Methyltransferase Expression in Pulmonary Large‐Cell Neuroendocrine Carcinoma: Pilot Experimental Data Targeted DNMT1, DNMT3A, and DNMT3B
Source: Cancer Rep (Hoboken). 2026 Mar 19;9(3):e70513. doi: 10.1002/cnr2.70513 (PMC13093775; doi:10.1002/cnr2.70513)

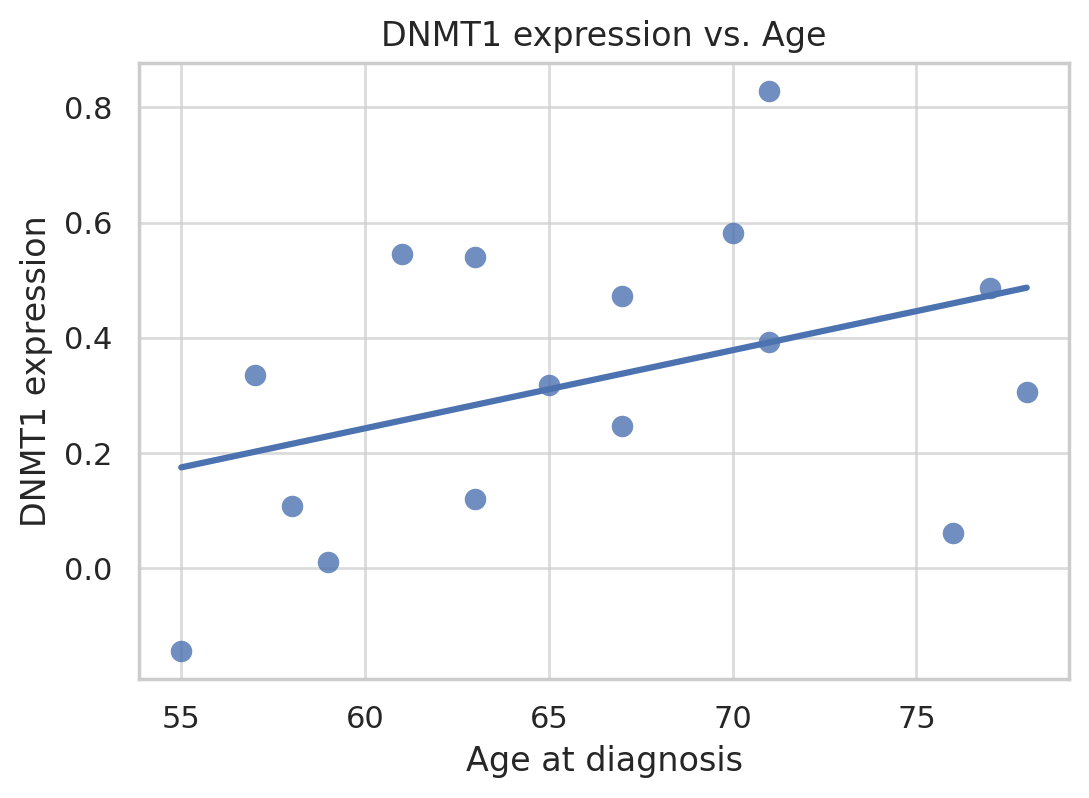

Supplement: Supplementary file 1 — Figure S5. Scatterplot of DNMT1 expression versus patient age. A non‐significant positive correlation was observed, suggesting a trend toward higher DNMT1 expression with increasing age. [file CNR2-9-e70513-s004.tif]

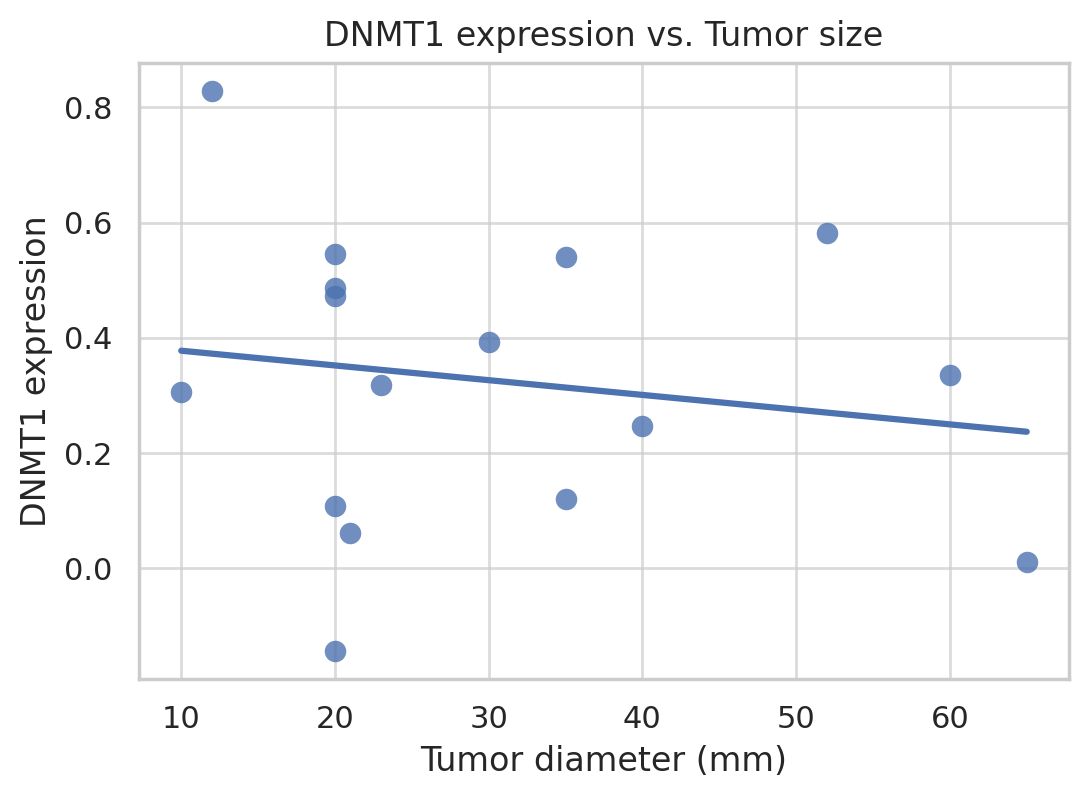

Supplement: Supplementary file 2 — Figure S6. Scatterplot of DNMT1 expression versus tumor diameter. No correlation was found between DNMT1 expression levels and tumor size. [file CNR2-9-e70513-s003.tif]

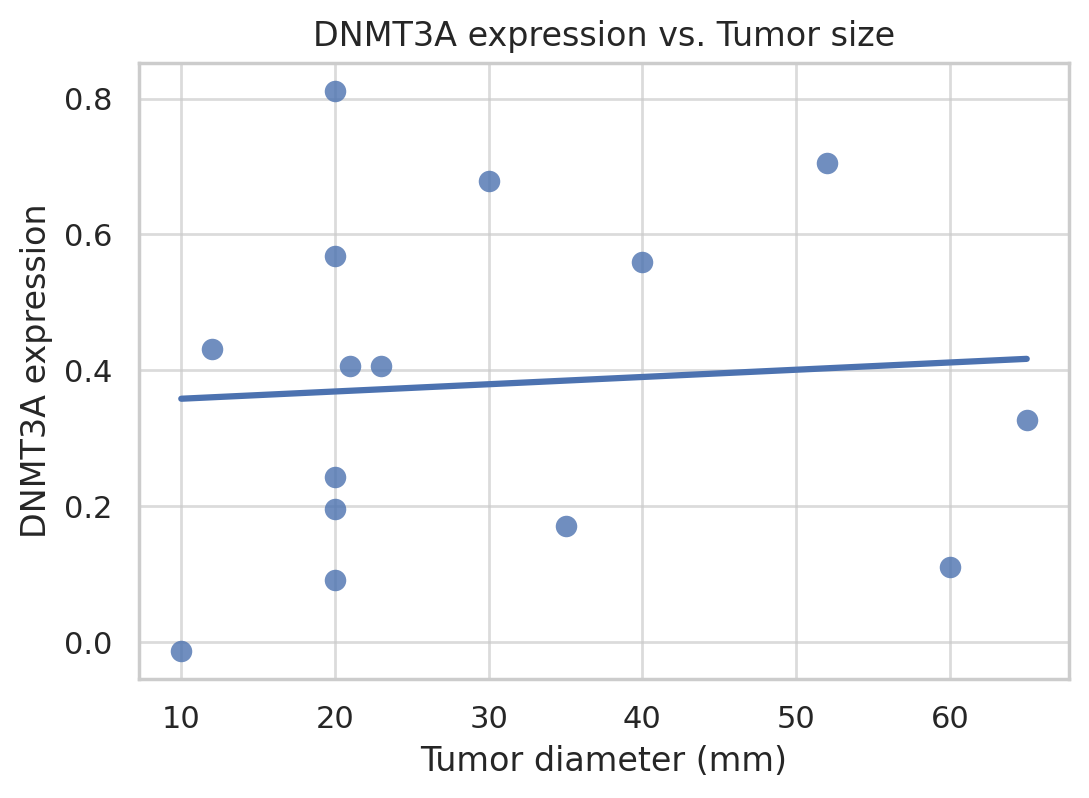

Supplement: Supplementary file 3 — Figure S7. Scatterplot of DNMT3A expression versus patient age. No correlation was observed between DNMT3A expression levels and patient age (Pearson r = −0.09, p = 0.75). [file CNR2-9-e70513-s001.tif]

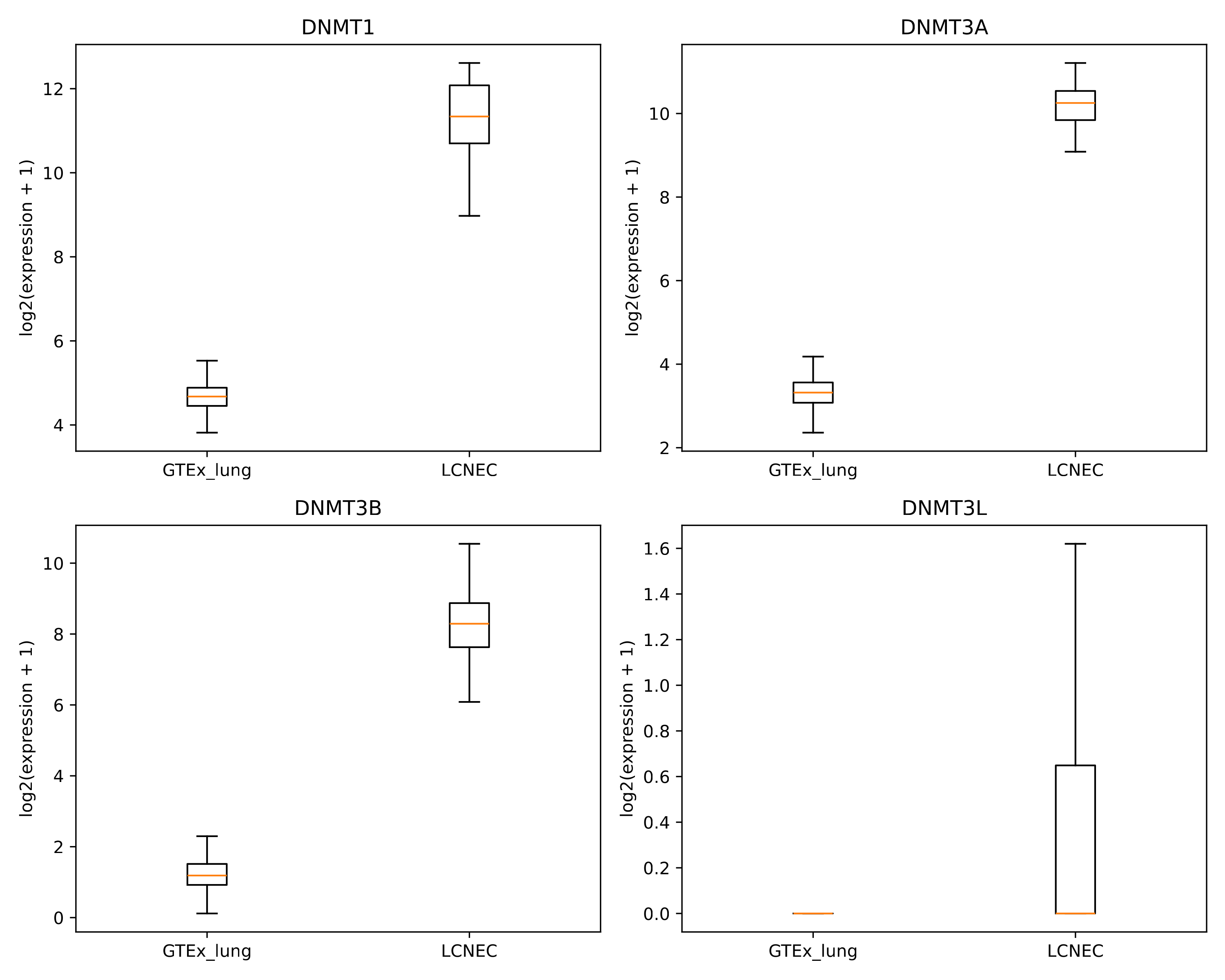

Supplement: Supplementary file 5 — Figure S9. Expression distributions of DNMT1, DNMT3A, DNMT3B, and DNMT3L in LCNEC (George et al. 2018; RNA‐seq, RSEM expected counts; n = 66) versus normal lung (GTEx v10; TPM; n = 604). Values were log2(x + 1) transformed; boxes show IQR with median; whiskers depict 1.5 × IQR, outliers hidden. Because of different pipelines (RSEM vs. TPM), this comparison is descriptive. [file CNR2-9-e70513-s002.tif]

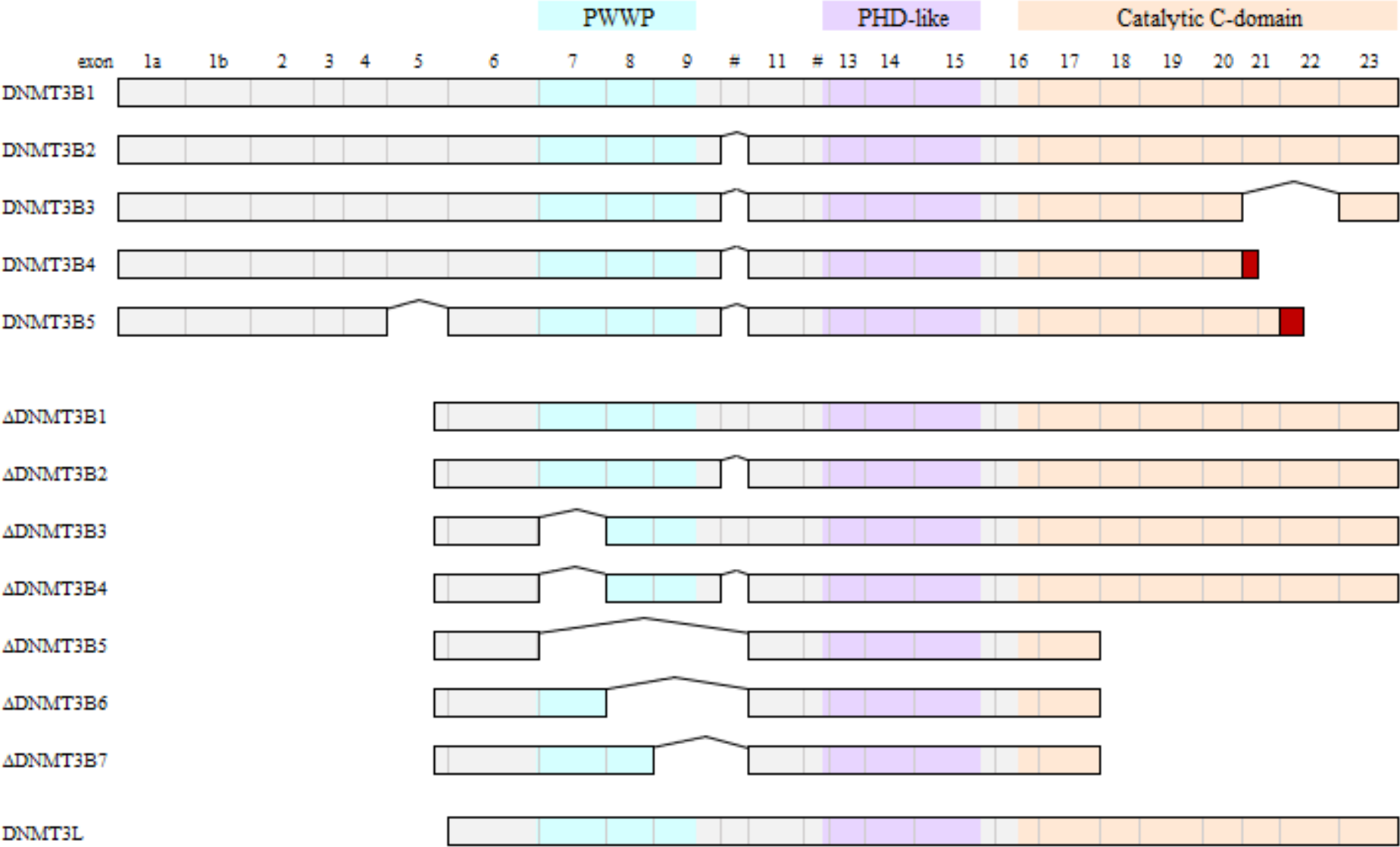

Supplement: Supplementary file 6 — Figure S10. Schematic diagram of some members DNMT3B (3B1, 3B2, 3B3, 3B4, 3B5) and ΔDNMT3B (Δ3B1, Δ3B2, Δ3B3, Δ3B4, Δ3B5, Δ3B6, Δ3B7) subfamilies, and DNMT3L (A) and a comparison their transcripts (B). Figure adapted from Gujar et al. 2019, Genes, with modifications [29]. DNMT3B/3 L—DNA methyltransferase 3B/3 L. [file CNR2-9-e70513-s006.zip › S.10A.tif]

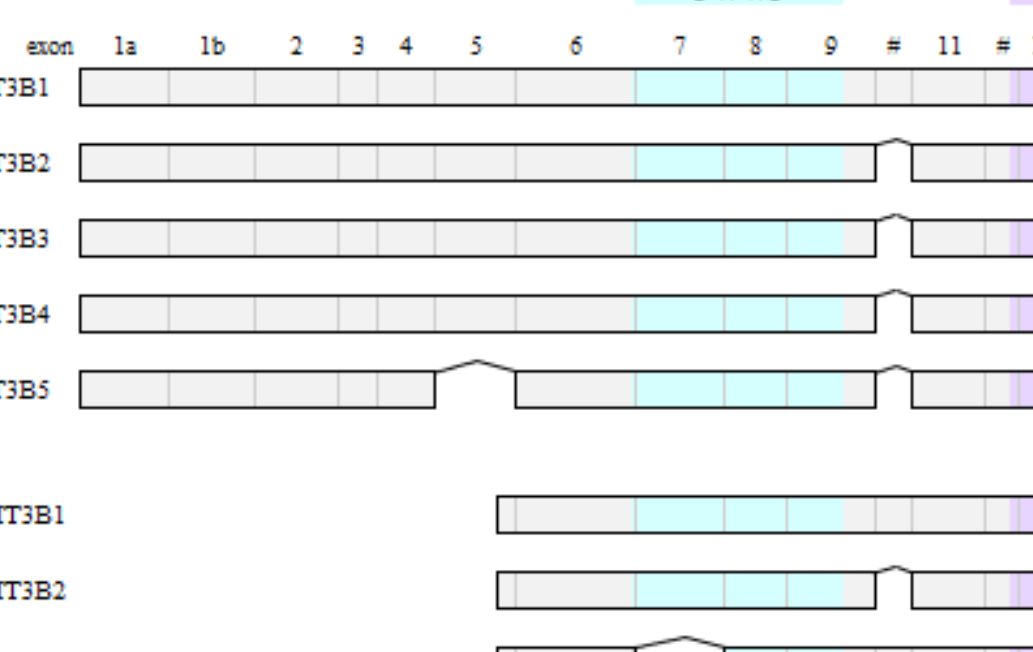

Supplement: Supplementary file 6 — Figure S10. Schematic diagram of some members DNMT3B (3B1, 3B2, 3B3, 3B4, 3B5) and ΔDNMT3B (Δ3B1, Δ3B2, Δ3B3, Δ3B4, Δ3B5, Δ3B6, Δ3B7) subfamilies, and DNMT3L (A) and a comparison their transcripts (B). Figure adapted from Gujar et al. 2019, Genes, with modifications [29]. DNMT3B/3 L—DNA methyltransferase 3B/3 L. [file CNR2-9-e70513-s006.zip › S.10B.tif]
