# Supplementary material for: Altered DNA Methyltransferase Expression in Pulmonary Large‐Cell Neuroendocrine Carcinoma: Pilot Experimental Data Targeted DNMT1, DNMT3A, and DNMT3B
Source: Cancer Rep (Hoboken). 2026 Mar 19;9(3):e70513. doi: 10.1002/cnr2.70513 (PMC13093775; doi:10.1002/cnr2.70513)
Supplement: Supplementary file 7 — Table S1: Clinicopathological features of the 18 cases of LCNEC. Table S2: Table of primers used for quantitative Real‐time PCR. Table S3: Mean ΔCq values with 95% confidence intervals (CI) for DNMT1, DNMT3A, and DNMT3B expression level relative to the calibrator sample. Values represent point estimates (mean differences) against the calibrator. Table S4: Summary of DNMT1, DNMT3A, and DNMT3B expression and their associations with clinicopathological parameters in lung cancer patients. Patients were categorized as having upregulated expression (> 0) or downregulated expression (< 0) of each gene. Values are shown as counts, percentages, means, or correlation coefficients (Pearson's r, p‐values). Overexpression predominated for DNMT1 and DNMT3A, while DNMT3B displayed a balanced distribution. No statistically significant correlations between gene expression and clinicopathological parameters were observed, although DNMT1 expression showed a weak, non‐significant trend toward higher levels with increasing age. [file CNR2-9-e70513-s007.docx]

**Supplementary table 1.**

| Primers compatible with DNMT1 probe | |
| --- | --- |
| Forward | 5´–CAAACCCCTTTCCAAACCTC–3´ |
| Reverse | 5´–TAATCCTGGGGCTAGGTGAA– 3´ |
| Primers compatible with DNMT3A probe | |
| Forward | 5´–CCTGAAGCCTCAAGAGCAGT–3´ |
| Reverse | 5´–TGGTCTCCTTCTGTTCTTTGC–3´ |
| Primers compatible with DNMT3B probe | |
| Forward | 5´–GGAAATTAGAATCAAGGAAATACGA–3´ |
| Reverse | 5´–AATTTGTCTTGAGGCGCTTG–3´ |

**Supplementary table 2.**

|  | **DNMT1** | **DNMT3A** | **DNMT3B** |
| --- | --- | --- | --- |
| Patients (n) | 17 | 16 | 6 |
| Upregulated expression | 16 | 15 | 3 |
| Downregulated expression | 1 | 1 | 3 |
| Mean age (OE) | 66.9 | 65.5 | 61.7 |
| Mean tumor size (OE, mm) | 30.9 | 31.3 | 35.7 |
| Recurrence in OE (%) | 4/16 (25%) | 4/15 (26.7%) | 1/3 (33.3%) |
| Recurrence in UE (%) | 0/1 (0%) | 0/1 (0%) | 2/3 (66.7%) |
| Correlation with age (r, p) | 0.39, 0.13 | -0.09, 0.75 | -0.17, 0.74 |
| Correlation with tumor size (r, p) | -0.17, 0.53 | 0.07, 0.79 | 0.01, 0.98 |
| Correlation with inflammation (r, p) | 0.05, 0.86 | -0.14, 0.62 | 0.21, 0.69 |

**Supplementary Table 3.**

| Gene | n | Mean ΔCq | 95% CI |
| --- | --- | --- | --- |
| DNMT1 | 17 | 0.1508 | [0.0796, 0.2221] |
| DNMT3A | 16 | 0.0256 | [0.01, 0.0412] |
| DNMT3B | 13 | 0.056 | [-0.0423, 0.1543] |

**Supplementary Table 4**.

| Gene | Variable | n | Effect size | 95% CI | p-value |
| --- | --- | --- | --- | --- | --- |
| DNMT1 | Age | 17 | 0.39 | [-0.11, 0.73] | 0.121 |
| DNMT1 | Tumor size | 17 | -0.17 | [-0.62, 0.35] | 0.537 |
| DNMT3A | Age | 16 | -0.08 | [-0.55, 0.43] | 0.774 |
| DNMT3A | Tumor size | 16 | 0.08 | [-0.43, 0.56] | 0.763 |
